# The anti‐angiogenesis role of FBXW7 in diabetic retinopathy by facilitating the ubiquitination degradation of c‐Myc to orchestrate the HDAC2

**DOI:** 10.1111/jcmm.16204

**Published:** 2020-12-25

**Authors:** Lihua Hu, Xiangyun Lv, Dai Li, Wanping Zhang, Guangyao Ran, Qingchun Li, Jun Hu

**Affiliations:** ^1^ Aier Eye Hospital of Wuhan University Wuhan China; ^2^ School of Optometry Hubei University of Science and Technology Xianning China

**Keywords:** Angiogenesis, c‐Myc, Diabetic retinopathy, FBXW7, HDAC2, HIF‐1α, VEGF

## Abstract

Diabetic retinopathy (DR) is the most prevalently occurring microvascular complication in diabetic patients that triggers severe visual impairments. The anti‐angiogenesis role of FBXW7 has been identified in breast cancer. Therefore, this study intends to decipher the mechanism of FBXW7 in angiogenesis of DR. DR model was induced on mice using high‐glucose (HG) and high‐fat diet, and retinal microvascular endothelial cells (RMECs) isolated from normal mice were induced with HG, followed by evaluation of FBXW7, Ki67, HIF‐1α and VEGF expression by immunofluorescence, immunohistochemistry or Western blot analysis. After gain‐ and loss‐of‐function assays in normal and DR mice, angiogenesis was assessed by CD31 fluorescence staining and Western blot analysis. After ectopic expression and silencing experiments in HG‐induced RMECs, RMEC proliferation, migration and angiogenesis were, respectively, determined by EdU, Transwell and in vitro angiogenesis assays. The impact of FBXW7 on the ubiquitination of c‐Myc was studied by cycloheximide chase assay and proteasome inhibition, and the binding of c‐Myc to HDAC2 promoter by dual‐luciferase reporter gene experiment. DR mice and HG‐induced RMECs possessed down‐regulated FBXW7 and up‐regulated Ki67, HIF‐1α and VEGF. Silencing FBXW7 enhanced angiogenesis in normal mouse retinal tissue, but overexpressing FBXW7 or silencing c‐Myc diminished angiogenesis in DR mouse retinal tissue. Overexpressing FBXW7 or silencing c‐Myc depressed proliferation, migration and angiogenesis in HG‐induced RMECs. FBXW7 induced c‐Myc ubiquitination degradation, and c‐Myc augmented HDAC2 expression by binding to HDAC2 promoter. Conclusively, our data provided a novel sight of anti‐angiogenesis role of FBXW7 in DR by modulating the c‐Myc/HDAC2 axis.

## 1 INTRODUCTION

Diabetes is reported to cause metabolic change, vascular structural change and chronic inflammation, which may eventually lead to loss of retinal ganglion cell and impair visual function.^1^ Statistics depict that diabetic retinopathy (DR) is perceived as the leading cause of vision loss among adults aged 20‐74 years and such kind of disorder can be commonly seen in middle‐income and high‐income area.^2^ The whole course of DR is mainly classified into early non‐proliferative lesions and late proliferative lesions, and the late lesions have a great impact on the vision of patients.^3^ However, it is disappointing that there are few methods to treat DR, such as intravitreal vascular endothelial growth factor (VEGF) inhibitors ('anti‐VEGFs'), new steroids and laser photocoagulation.^4^ Therefore, the incidence of DR is increasing in worldwide and is rapidly becoming a major cause of a global epidemic and vision loss.^5^ The change of retinal microcirculation is the precursor of DR.^6^ This is due to retinal vascular damage and retinal blood barrier caused by diabetes.^7^, ^8^ Therefore, it is necessary to strengthen the research on the molecular mechanism of inhibiting angiogenesis DR so as to develop new therapeutic methods for DR.

F‐box and WD‐40 domain protein (FBXW7), one of the crucial identification factors of ubiquitin‐proteasome degradation pathway, can decrease many oncogene proteins, such as c‐Myc, cyclin E, Notch and Jun.^9^, ^10^ Moreover, a prior research evidenced the involvement of FBXW7 in retinal angiogenesis in mice.^11^ More importantly, another study also discovered that silencing FBXW7 induced angiogenesis of human retinal endothelial cells (hRECs) in DR.^12^ A previously reported research noted that c‐Myc could promote the proliferation of retinal pigment epithelial cells in rats.^13^ Also, it has been unravelled that c‐Myc assumed a contributory role in angiogenesis of endothelial cells.^14^ Interestingly, it was noted in a prior study that c‐myc knock‐down resulted in alleviation of DR progression in vivo by accelerating the release of Müller cell‐derived pro‐inflammatory cytokines.^15^ Specifically, c‐Myc can bind to histone deacetylase‐2 (HDAC2) promoter to promote cell proliferation.^16^ Notably, the pivotal involvement of HDAC2 has been uncovered in for beta‐adrenergic signalling‐induce angiogenesis in prostate cancer.^17^ Additionally, the proliferation of vascular endothelial cells leading to angiogenesis is considered to be a major driving process of DR.^18^ Moreover, it has been demonstrated that VEGF played critical role in muller glia viability and could exert protective effect on neurons in DR.^19^ From the above, we speculated that the FBXW7/c‐Myc/HDAC2 axis participated in the angiogenesis of DR via VEGF. Therefore, we employed molecular biology and biochemical methods to implemented relevant experiments to study the regulation of FBXW7‐mediated ubiquitination degradation of c‐Myc on the HDAC2/VEGF axis to inhibit the angiogenesis of DR.

## 2 MATERIALS AND METHODS

### 2.1 Ethics statement

Animals were separately housed in specific pathogen‐free facilities during the experiment. The experiments were implemented with the ratification from the Biomedical Research Ethics Committee of Wuhan University and strictly following the recommendations in the laboratory animal care and use guidelines published by the National Institutes of Health. Experimental procedures complied with the Association for Research in Vision and Ophthalmology guidelines regarding the use of animals in ophthalmology and vision studies.

### 2.2 Study subjects

Totally, 48 healthy adult male C57Bl/6J mice (weighing 20.90 ± 2.05 g; aged 5 weeks) were attained from Experimental Animal Center (Chinese Academy of Sciences, Shanghai, China). Among them, 18 mice were fed with normal diet, and the remaining 30 mice were fed with high‐glucose and high‐fat diet (66.5% maintenance feed + 10% lard + 20% sucrose + 2.5% cholesterol + 1% sodium cholate) for DR model induction. After 6 weeks, each DR mouse was intraperitoneally injected with 0.45% streptozotocin (45 mg/kg, dissolved in 0.1 mol/L sterile citrate buffer [pH = 4.5], S0130‐50MG, Sigma, St. Louis, MO, USA). Control mice were injected intraperitoneally with an equivalent dose of sterile citrate buffer (pH = 4.5). All mice were housed with a 12‐hour light/dark cycle and free access to food and water. After 72‐h injection, tail vein blood was harvested to detect blood glucose levels in mice. Mice with blood glucose levels ≥ 16.7 mmol/L were considered as diabetic mice. After the diabetic mice were fed with the high‐glucose and high‐fat diet for 4 weeks, the blood glucose concentration was measured again. Mice that were stable for at least 5 days with a blood glucose concentration higher than 16.7 mmol/L were selected as DR mice. DR mice were further fed with high‐glucose and high‐fat diet. The control mice were fed with normal feed. After 12 weeks of modelling (17‐week mice), all mice were euthanized after intraperitoneal injection of 2% pentobarbital sodium for anaesthesia, and the eyes were quickly and accurately attained. All the retinal tissues were vitreous without the lens. One part of the tissue was fixed with 4% paraformaldehyde, and paraffin‐embedded slices were made for subsequent histological analysis. The other part was preserved at −80ºC for following experiments. Normal diet‐fed mice were untreated (control group), or treated with short hairpin RNA (sh)‐negative control (NC) (sh‐NC group) or sh‐FBXW7 (shi‐FBXW7 group), whereas DR mice were untreated (DR group), or treated with sh‐NC (DR + sh‐NC group), overexpression (oe)‐NC (DR + oe‐NC group), oe‐FBXW7 (DR + oe‐FBXW7 group) or sh‐c‐Myc (DR + sh‐c‐Myc group) (n = 6 mice/group).

### 2.3 Intravitreal injection

Subsequent to anaesthesia of mice by intraperitoneal injection of 2% pentobarbital sodium, type II adeno‐associated virus (AAV, 1 × 10^12^ μg/mL, 2 μL) containing sh‐NC, sh‐FBXW7, oe‐NC or oe‐FBXW7 was injected into the vitreous cavity. The eyes were dissected after one month.

### 2.4 Immunohistochemistry

The 5‐μm paraffin‐embedded slices were made. Subsequent to fixing, dehydration, baking, dewaxing and hydration, the slices were repaired with 0.1 M sodium citrate, boiled for 20 min, naturally cooled and then inactivated with 3% catalase for 15 min. The slices were blocked by 5% bovine serum albumin (BSA) before 30‐min incubation at ambient temperature. Next, the overnight slice incubation was implemented with primary antibodies (1:500, Abcam, Cambridge, UK) to rabbit anti‐FBXW7 (ab109617), rabbit anti‐c‐Myc (ab32072), rabbit anti‐Ki67 (ab197234), mouse anti‐hypoxia‐inducible factor‐1 alpha (HIF‐1α) (ab1) and mouse anti‐VEGF (ab1316) in a humidified box at 4ºC. After drying, biotinylated goat anti‐rabbit immunoglobulin G (IgG; 1:1000, ab6721, Abcam) was supplemented to the slices at 37ºC for 30‐min incubation. The horseradish peroxidase (HRP)‐labelled streptavidin protein working solution (DA1010, Solarbio, Beijing, China) was added dropwise into the slices and positioned at 37ºC for 20 min. Diaminobenzidine was adopted for colour development, and the time depended on the actual situation. Subsequent to haematoxylin counterstaining, the slices were returned to blue with 1% ammonia. Following dehydration, clearing and sealing, the slices were observed using an optical microscope (XSP‐36, Bosda, Shenzhen, China). Five random high‐power fields (× 200) were chosen for each slice, with 100 cells counted in each field. The number of positive cells < 5% was considered as negative, and the positive cells ≥ 5% were positive. The results of immunohistochemistry were independently scored by two persons. Five fields of view at × 200 magnification were randomly captured for each replicate using an optical microscope (XSP‐36, Bosda).

### 2.5 Immunofluorescence

The sections were blocked with goat serum for 15 min at ambient temperature and then incubated with rabbit polyclonal antibodies (1:100; Abcam) to FBXW7 (ab109617) and CD31 (ab24590) at 4°C overnight. The 60‐min further section culture was performed with fluorescent‐labelled secondary goat anti‐rabbit IgG H&L antibody (1:1000, ab150077, Abcam) at ambient temperature. Subsequent to nuclei staining with 4', 6‐diamidino‐2‐phenylindole (DAPI) (C0065, Solarbio), the sample was photographed under a fluorescence microscope (FM‐600, Pudan Optical, Shanghai, China) at a magnification of 400‐fold.

### 2.6 Reverse transcription quantitative polymerase chain reaction (RT‐qPCR)

Subsequent to total RNA isolation using TRIzol (15 596 026, Invitrogen, Carlsbad, California, USA), cDNA was generated from mRNA in the light of manuals of a commercially available kit (RR047A, Takara, Tokyo, Japan). cDNA was subject to RT‐qPCR using SYBR Premix Ex Taq II (Perfect Real Time) kit (DRR081, Takara) on the ABI 7300 instrument (Applied Biosystems, Foster City, CA, USA), with each reaction run in triplicate. Results were calculated by using the 2^‐△△CT^ method and standardized by glyceraldehyde‐3‐phosphate dehydrogenase (GAPDH). The primer information is listed in Table 1.

**Table 1:** qRT‐PCR primer sequences used in this study

Gene	Sequence (5’‐ 3’)
FBXW7	Forward: 5’‐GAAAGTTGGACCATGGTTCTGAAG‐3’
	Reverse: 5’‐CCAGCAACTTCTCTGGTCCG‐3’
c‐Myc	Forward: 5’‐GAAACACAAACTCGAACAGC‐3’
	Reverse: 5’‐TGAAGCTTACAGTCCCAAAG‐3’
GAPDH	Forward: 5’‐CAATGTGTCCGTCGTGGATCT‐3’
	Reverse: 5’‐GTCCTCAGTGTAGCCCAAGATG‐3’

### 2.7 Protein extraction and quantification

Subsequent to protein extraction using protease inhibitor‐contained radio‐immunoprecipitation assay buffer (Boster, Wuhan, China), protein concentration was estimated using a bicinchoninic acid protein quantification kit (Boster). The protein sample was separated using freshly prepared 10% sodium dodecyl sulphate‐polyacrylamide gel electrophoresis before transferring onto polyvinylidene fluoride membranes. Afterwards, overnight membrane incubation was conducted with primary antibodies (1:1000, Abcam) to rabbit anti‐FBXW7 (ab109617), rabbit anti‐c‐Myc (ab32072), rabbit anti‐Ki67 (ab197234), mouse anti‐HIF‐1α (Ab1), murine anti‐VEGF (ab1316) and murine anti‐GAPDH (ab8245) at 4ºC. Then, HRP‐labelled secondary goat anti‐rabbit (ab205719) or goat anti‐mouse (ab205719) antibodies (1:2000, Abcam) were added for 1‐h incubation at 37ºC. Electrogenerated chemiluminescence (ECL) working solution (EMD Millipore, Billerica, MA, USA) was supplemented to the member before 1‐min incubation at 37ºC. Subsequent to the removal of the excess ECL reagent, the blot was visualized by X‐ray in the dark for 5‐10 min and developed. The grey value of target protein bands was quantified using ImageJ software, with GAPDH utilized for normalization. Each experiment was repeated 3 times.

### 2.8 Isolation and identification of primary retinal microvascular endothelial cells (RMECs)

Five normal mice were selected and anesthetized by intraperitoneal injection with 2% pentobarbital sodium. After harvesting of the eyeballs, dissection was made along with the cornea under a stereomicroscope. The vitreous body, lens and cornea were bluntly separated, ground and filtered through a 200‐mesh filter. The tissue was obtained into a 15‐mL centrifuge tube before detachment with 3 mL trypsin encompassing 0.1% ethylene diamine tetraacetic acid. The specimens were warmed for 3‐5 min by water bath at 37ºC, followed by 5‐min centrifugation at 800 rpm. Subsequent to removal of the trypsin, the specimens were water‐bathed with 0.5% collagenase II for 30‐min and filtered through a 300‐mesh filter. The attained cell suspension was centrifuged at 800 rpm for 5 min. After discarding of the supernatant, RMEC culture was implemented in Dulbecco's modified Eagle's medium encompassing 10% foetal bovine serum (FBS), 100 µg/mL streptomycin, 50 µg/mL penicillin, 1% endothelial cell growth supplement and 50 µg/mL heparin in a 37ºC incubator with, 5% CO_2_ and 95% humidity. The purity of the isolated RMECs was determined by VEGF staining, and the following experiments were conducted with the RMECs with purity above 90%.

### 2.9 Cell culture and transient transfection

When the growth density reached about 80%, cells were passed to the next generation. RMECs were treated with 25 mmol/L glucose as the high‐glucose group, and RMECs were treated with 2.5 mM glucose as controls. Subsequent to culture in a six‐well plate at 3 × 10^5^ cells/well, infection was implemented on the 50% confluent RMECs. Silencing lentiviral vectors, overexpressed lentiviral vectors and corresponding NC lentiviral vectors were attained from GeneChem (Shanghai, China). The silencing sequences are described in Table 2.

**Table 2:** Interference sequence used in this study

Gene	Sequence (5’‐3’)
FBXW7	sh‐RNA‐1: CCACGTTAGAATCTGTGACAT
	sh‐RNA‐2: CCAGAGACTGAAACCTGTCTA
	sh‐RNA‐3: CGCATAGTTAGTGGTTCTGAT
c‐Myc	sh‐RNA‐1: GCGACGAGGAAGAGAATTTCT
	sh‐RNA‐2: GCTCTGCTCTCCATCCTATGT
	sh‐RNA‐3: GGAAACGACGAGAACAGTTGA

### 2.10 5‐Ethynyl‐2’‐Deoxyuridine (EdU) labelling assay

The cells were cultured in a 24‐well plate, with three replicate wells for each group. Subsequent to supplementation of EdU into the medium to reach 10 μmol/L, 2‐h cell incubation was implemented in an incubator. Following medium removal, 15‐min cell fixing was carried out with 4% paraformaldehyde. The 20‐min cell incubation was conducted in phosphate buffered saline (PBS) encompassing 0.5% Triton 100 at 37ºC before 30‐min staining with 100 µL staining solution in each well at ambient temperature in dark. Subsequent to 5‐min DAPI staining, the number of positive cells in 6‐10 random fields was recorded with a fluorescence microscope (FM‐600, Pudan Optical). EdU labelling rate (%) was calculated as the number of positive cells/(number of positive cells + number of negative cells) × 100%. Each experiment was repeated 3 times.

### 2.11 Transwell migration assay

Subsequent to 12‐h cell incubation in medium without serum, the harvested cells were resuspended in medium without serum (1 × 10^5^ cells/mL). The medium encompassing 10% FBS was supplemented to the lower chamber. Cell suspension (100 µL) was supplemented to the Transwell chamber before 24‐h culture at 37ºC. Cells that did not invade the surface of the Matrigel membrane was gently removed with a cotton swab. The remaining cells were fixed with 100% methanol before 1% crystal violet (Sigma) staining. The stained cells were counted under an inverted light microscope (Carl Zeiss, Jena, Germany) in random 5 fields. Each experiment was repeated 3 times.

### 2.12 Angiogenesis assay in vitro

The Matrix gel (M8370, Solarbio) was attained at −20ºC the day before use and thawed at 4ºC overnight. Meanwhile, 96‐well plates, pipette tips and other instruments used in the experiment were stored at −20ºC. Matrix gel was supplemented to the 96‐well plates (70 µL/well) which were positioned in a 37ºC incubator for 1 h. After the basement gel solidified, virus‐infected cells were trypsinized and counted. The infected RMECs were seeded at 5 × 10^4^ cells/well onto a pre‐coated base plate. Three replicates were made for each group. Four fields of view per well were observed under a phase‐contrast microscope, and the number of small tubes was counted and imaged.

### 2.13 Dual‐luciferase reporter gene experiment

The site in which c‐Myc bound to the HDAC2 promoter was analysed using the PROMO (http://alggen.lsi.upc.es/cgi-bin/promo_v3/promo/promoinit.cgi?dirDB=TF_8.3). The HDAC2 promoter segment encompassing wild‐type (WT) or mutant type (MUT) of binding site to c‐Myc was constructed into upstream of F‐Luciferase in the pGL3‐Basic vector. The vectors were co‐transfected with c‐Myc into 293T cells. Analysis of F‐Luciferase activity reflected the level of promoter activity. Subsequent to 48‐h transfection, the attained cells were lysed, followed by luciferase activity detection on a dual‐luciferase reporter system (GeneChem). The R‐Luciferase vector was used as an internal reference to calculate the ratio of the Firefly luciferase and the Renilla luciferase, therefore revealing the activation of the target reporter gene.

### 2.14 Statistical analysis

Data were described as the mean ± standard deviation. Statistical comparisons were performed using unpaired t test when only two groups were compared or by Tukey's test‐corrected one‐way analysis of variance (ANOVA) when more than two groups were compared. All statistical analyses were completed with SPSS 21.0 software (IBM Corp. Armonk, NY, USA), with *P* < .05 as a level of statistical significance.

## 3 RESULTS

### 3.1 FBXW7 was underexpressed, while Ki67, HIF‐1α and VEGF were overexpressed in retinal tissue of DR mice

**Figure 1:**
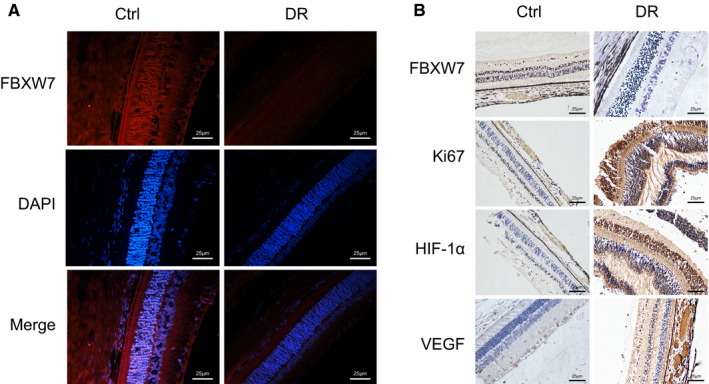
FBXW7 is highly expression but Ki67, HIF‐1α and VEGF are poorly expressed in the retinal tissue of DR mice. (A) Immunofluorescence detection of FBXW7 expression in retinal tissues of control mice (n = 6) and DR mice (n = 6) (scale: 25 μm). (B) Immunohistochemical detection of FBXW7, Ki67, HIF‐1α and VEGF expression in retinal tissues of control mice (n = 6) and DR mice (n = 6) (scale: 25 μm)

To study the role of FBXW7 in DR, we first induced a DR model on mice. As reflected by immunofluorescence (Figure 1A), in contrast to control mice, FBXW7 in retinal tissue of DR mice was significantly down‐regulated (Figure 1A). Researchers have reported that HIF‐1α promotes angiogenesis in DR by up‐regulating VEGF.^20^ In our case, immunohistochemistry demonstrated that compared with control mice, the positive expression rate of FBXW7 was obviously reduced, whereas the positive expression rates of angiogenesis‐related factors Ki67, HIF‐1α and VEGF were potently increased in retinal tissue of DR mice (Figure 1B). These results suggested that FBXW7 was down‐regulated while Ki67, HIF‐1α and VEGF were up‐regulated in the retinal tissue of DR mice.

### 3.2 Silencing FBXW7 promoted angiogenesis in the retina of normal mice

**Figure 2:**
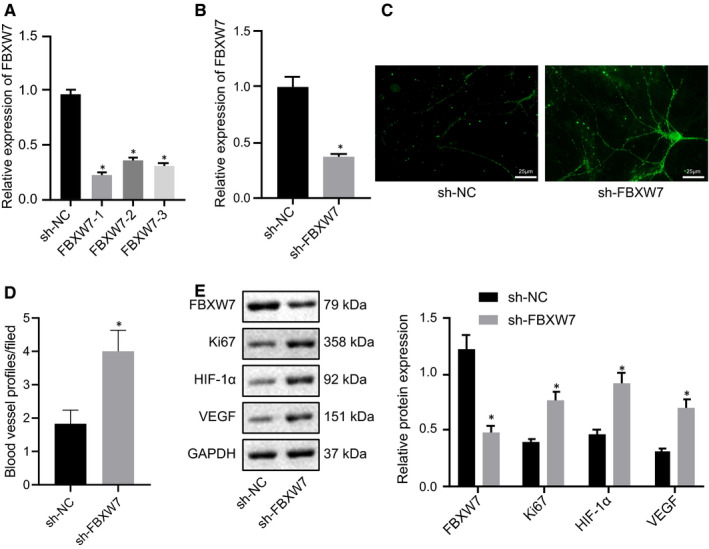
Silencing FBXW7 in normal mouse retina promotes angiogenesis. (A) RT‐qPCR identification of the silencing efficiency of FBXW7 in RMECs. * *P* < .05 *vs*. sh‐NC‐treated RMECs. Normal diet‐fed mice were treated with sh‐NC or sh‐FBXW7. (B) RT‐qPCR detection of the silencing efficiency of sh‐FBXW7 in the retina of normal diet‐fed mice. (C) Representative image of CD31 fluorescence staining of intraretinal angiogenesis in the retina of normal diet‐fed mice (Arrows indicated blood vessel profiles [BVPs], scale: 25 μm.). (D) Quantitative results of panel C. E, Western blot analysis of FBXW7, HIF‐1α, Ki67 and VEGF expression in the retina of normal diet‐fed mice. * *P* < .05 *vs*. normal diet‐fed mice treated with sh‐NC. The measurement data were expressed as mean ± standard deviation. The unpaired t test was used for comparison between two groups. The cell experiments were repeated 3 times. n = 6 mice/group

Next, we evaluated the correlation between FBXW7 and angiogenesis by silencing FBXW7 in the retina of normal mice. Three sh‐FBXW7 sequences were designed, and RT‐qPCR found that decline of FBXW7 expression was caused in RMECs after sh‐FBXW7 treatment, among which the silencing efficiency of sh‐FBXW7‐1 was the most obvious. Therefore, the following experiments were implemented with sh‐FBXW7‐1 (Figure 2A). After normal mice were injected with AAV encompassing sh‐NC or sh‐FBXW7, RT‐qPCR showed that FBXW7 expression after sh‐FBXW7 treatment was strikingly reduced (Figure 2B). The results of CD31 fluorescence staining displayed that intraretinal angiogenesis in normal mice was potently elevated by silencing FBXW7 (Figure 2C and D). For Western blot analysis, FBXW7 silencing notably diminished FBXW7 expression but elevated Ki67, HIF‐1α and VEGF expression in retinal tissues of normal mice (Figure 2E). Therefore, FBXW7 silencing promoted angiogenesis in normal mice.

### 3.3 Overexpression of FBXW7 inhibited angiogenesis in retina of DR mice

**Figure 3:**
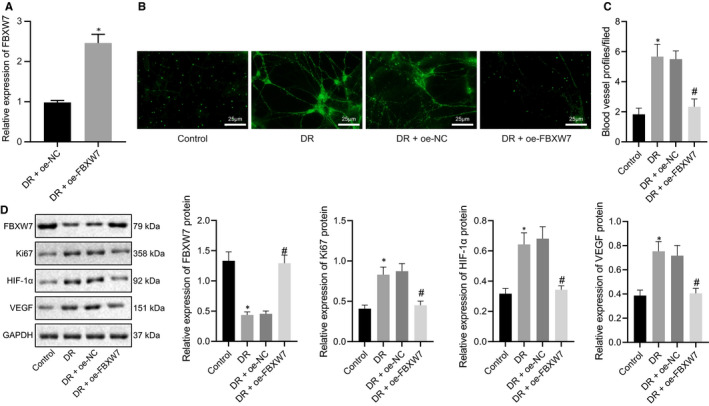
Overexpression of FBXW7 inhibits intraretinal angiogenesis in DR mice. (A) RT‐qPCR detection of the overexpression efficiency of FBXW7 in mice. * *P* < .05 *vs*. oe‐NC‐treated DR mice. Mice were fed with normal diet as control, whereas DR mice were untreated, or treated with oe‐NC or oe‐FBXW7. (B) Representative image of CD31 fluorescence staining of intraretinal angiogenesis (Arrows indicate blood vessel profiles [BVPs], scale: 25 μm.). (C) Quantitative results of panel B. (D) Western blot analysis of FBXW7, HIF‐1α, Ki67 and VEGF expression in mouse retina. * *P* < .05 *vs*. normal diet‐fed mice; # *P* < .05 *vs*. DR mice treated with oe‐NC. The measurement data were expressed as mean ± standard deviation. The unpaired t test was used for comparison between two groups, and Tukey's test‐corrected one‐way ANOVA was used for comparison among multiple groups. n = 6 mice/group

To further study the role of FBXW7 in the angiogenesis of DR mice, we overexpressed FBXW7 in retina of DR mice. RT‐qPCR manifested that FBXW7 expression was remarkably enhanced in retina of DR mice by oe‐FBXW7 (Figure 3A), in line with experimental requirements. CD31 fluorescence staining described that compared with control mice, blood vessel profiles increased, a large number of RMECs broke through the inner limit membrane into the vitreous cavity with abnormal arrangement, and retinal angiogenesis was accelerated in retina of DR mice. Overexpression of FBXW7 improved the retinal vascular profiles and reduced intraretinal angiogenesis in retina of DR mice (Figure 3B, C). Additionally, Western blot analysis illustrated that FBXW7 expression in retina was lower but HIF‐1α, Ki67 and VEGF expression was higher in DR mice than in control mice. On the contrary, overexpressing FBXW7 led to opposite trends in DR mice (Figure 3D). These data demonstrated that overexpression of FBXW7 inhibited retinal angiogenesis in DR mice.

### 3.4 Overexpression of FBXW7 reduced HG‐induced proliferation, migration and luminal formation of RMECs

**Figure 4:**
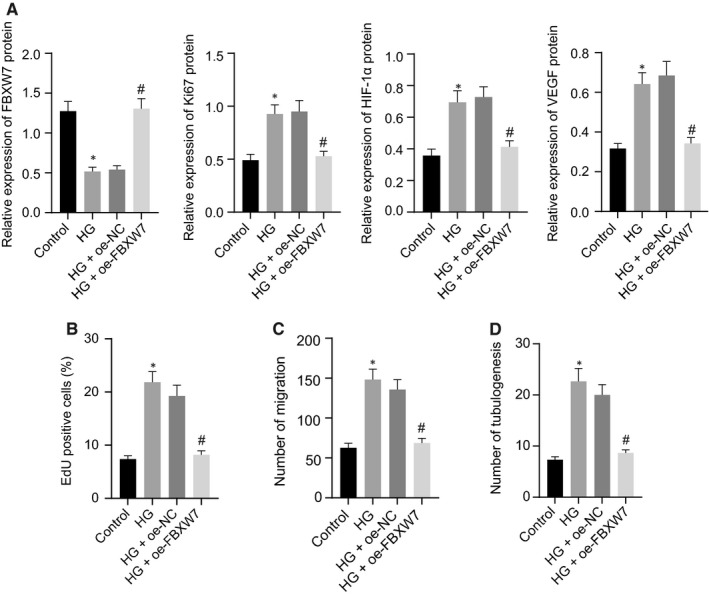
Overexpression of FBXW7 reduces HG‐induced proliferation, migration and angiogenesis of RMECs. NG‐treated RMECs were used as controls, whereas HG‐treated RMECs were uninfected, or infected with oe‐NC or oe‐FBXW7 lentivirus. (A) Western blot analysis of FBXW7, Ki67, HIF‐1α and VEGF expression in RMECs. (B) EdU assay of RMEC proliferation. (C) Transwell assay of RMEC migration. (D) Angiogenesis assay in vitro to identify the tube formation of RMECs. * *P* < .05 *vs*. NG‐treated RMECs; # *P* < .05 *vs*. HG‐treated RMECs transfected with oe‐NC. The measurement data were expressed as mean ± standard deviation. Tukey's test‐corrected one‐way ANOVA was used for comparison among multiple groups. The experiments were repeated 3 times

To reveal the mechanism of FBXW7 affecting DR retinal angiogenesis, RMECs were isolated from normal mice for further research and induced with HG. Western blot analysis manifested that FBXW7 expression in RMECs was lowered by HG induction, whereas Ki67, HIF‐1α and VEGF expression was enhanced. Overexpression of FBXW7 resulted in down‐regulated Ki67, HIF‐1α and VEGF in HG‐induced RMECs (Figure 4A), which is consistent with the results of in vivo experiments. As for EdU, Transwell and angiogenesis assay in vitro, the proliferation, migration and luminal formation of RMECs were severely increased after HG treatment, which was reversed by oe‐FBXW7 (Figure 4B‐D). Conclusively, FBXW7 up‐regulation depressed proliferation, migration and luminal formation of HG‐induced RMECs.

### 3.5 FBXW7 accelerated ubiquitination degradation of c‐Myc in HG‐induced RMECs

**Figure 5:**
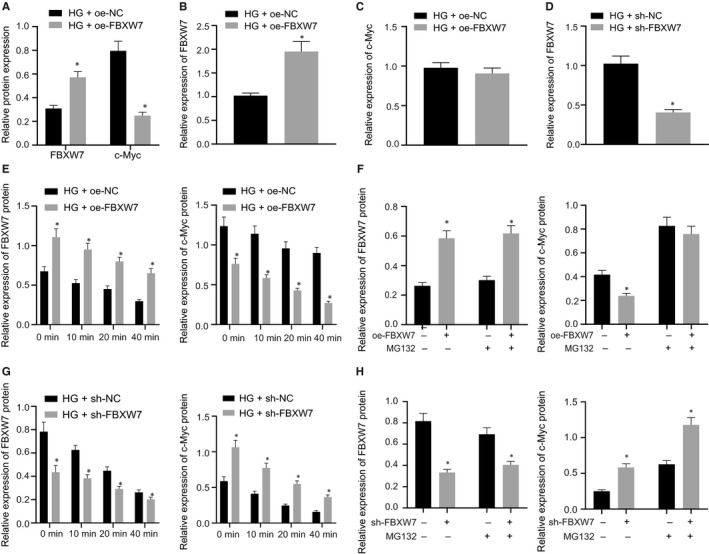
FBXW7 promotes c‐Myc ubiquitination degradation in HG‐induced RMECs. (A) Western blot analysis of FBXW7 and c‐Myc protein expression in HG‐induced RMECs after overexpression of FBXW7. (B) RT‐qPCR detection of FBXW7 mRNA level in HG‐induced RMECs after overexpression of FBXW7. (C) RT‐qPCR detection of the mRNA level of c‐Myc in HG‐induced RMECs after overexpression of FBXW7. *P < .05 *vs*. HG‐induced RMECs transfected with oe‐NC. (D) RT‐qPCR detection of the silencing efficiency of FBXW7 in HG‐induced RMECs. *P < .05 *vs*. HG‐induced RMECs transfected with sh‐NC. (E) Western blot analysis of FBXW7 and c‐Myc expression in HG‐induced RMECs after FBXW7 overexpression and different times of CHX (100 μg/mL) treatment. (F) Western blot analysis of FBXW7 and c‐Myc expression in HG‐induced RMECs after infection with oe‐NC or oe‐FBXW7 lentivirus and treatment with or without MG132 for 8 h. (G) Western blot analysis of the expression of FBXW7 and c‐Myc in HG‐induced RMECs after infection with sh‐NC or sh‐FBXW7 lentivirus and different times of CHX (100 μg/mL) treatment. (H) Western blot analysis of the expression of FBXW7 and c‐Myc in HG‐induced RMECs after infection with sh‐NC or sh‐FBXW7 lentivirus and treatment with or without MG132 for 8 h. * *P* < .05 *vs*. control treatment. The measurement data were expressed as mean ± standard deviation. The unpaired t test was used for comparison between two groups. The experiments were repeated 3 times

FBXW7 is a well‐known c‐Myc E3 ubiquitin ligase. An early research found that c‐Myc was highly expressed in DR, and that knocking down c‐Myc attenuated the progress of DR.^15^ Subsequent to FBXW7 overexpression in HG‐induced RMECs, RT‐qPCR and Western blot analysis depicted that FBXW7 mRNA and protein expression was augmented but c‐Myc protein expression was decreased in HG‐RMECs by oe‐FBXW7 treatment, along with unchanged c‐Myc mRNA level (Figure 5A‐C). Thus, we speculated that c‐Myc may be degraded by ubiquitination. To confirm that FBXW7 orchestrated c‐Myc ubiquitination, HG‐RMECs were infected with oe‐NC or oe‐FBXW7 lentivirus. Protein synthesis inhibitor cycloheximide (CHX) was adopted to inhibit protein synthesis. Western blot analysis exhibited that overexpression of FBXW7 contributed to enhancement of c‐Myc ubiquitination degradation (Figure 5E). When oe‐NC or oe‐FBXW7‐infected HG‐RMECs were treated with a proteasome inhibitor (MG132), we discovered that MG132 inhibited the ubiquitination degradation of c‐Myc caused by overexpression of FBXW7 (Figure 5F). In addition, we also infected RMECs with sh‐NC or sh‐FBXW7 lentivirus, and RT‐qPCR described that FBXW7 expression in HG‐induced RMECs was decreased by sh‐FBXW7 treatment (Figure 5D). Silencing FBXW7 significantly inhibited the ubiquitination degradation of c‐Myc (Figure 5G). Moreover, c‐Myc protein level increased significantly after silencing FBXW7 (Figure 5H). Therefore, FBXW7 suppressed c‐Myc through ubiquitin degradation in HG‐induced RMECs.

### 3.6 c‐Myc silencing reversed the HG‐induced angiogenesis of RMECs

**Figure 6:**
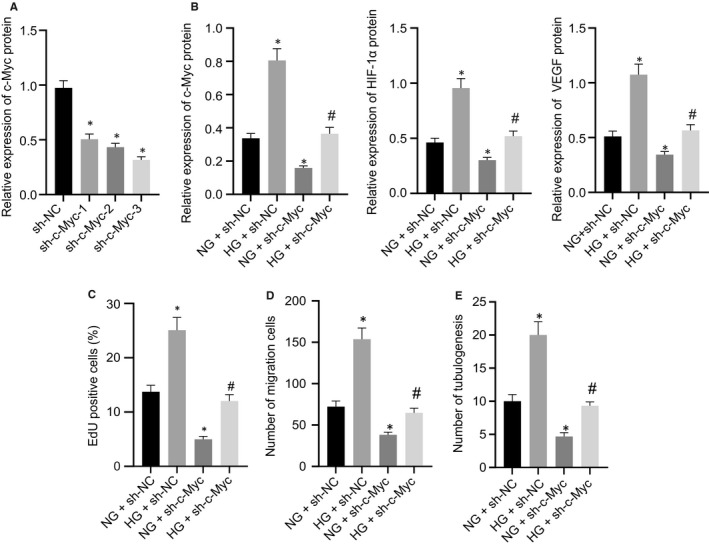
c‐Myc silencing rescues the increased angiogenesis of RMECs caused by HG. (A) Silencing efficiency of sh‐c‐Myc in RMECs. * *P* < .05 *vs*. RMECs infected with sh‐NC lentivirus. NG‐induced RMECs were infected with sh‐NC or sh‐c‐Myc lentivirus, whereas HG‐induced RMECs were infected with sh‐NC or sh‐c‐Myc lentivirus. (B) Western blot analysis of HIF‐1α and VEGF expression in RMECs. (C) EdU assay of RMEC proliferation. (D) Transwell assay to detect RMEC migration. (E) Angiogenesis assays in vitro to detect the tube formation of RMECs. * *P* < .05 *vs*. NG‐induced RMECs treated with sh‐NC; # *P* < .05 *vs*. HG‐induced RMECs treated with sh‐NC. The measurement data were expressed as mean ± standard deviation. Tukey's test‐corrected one‐way ANOVA was adopted for comparison among multiple groups. The experiments were repeated 3 times

To further confirm that FBXW7 repressed angiogenesis in DR by promoting c‐Myc ubiquitination degradation, three sh‐c‐Myc sequences were designed. As reflected by RT‐qPCR, reduction of c‐Myc expression was found in RMECs after treatment with all sh‐c‐Myc sequences, and sh‐c‐Myc‐3 had the highest silencing efficiency. Thus, sh‐c‐Myc‐3 was chosen for the following experimentation (Figure 6A). Western blot analysis showed the decreased c‐Myc, HIF‐1α and VEGF expression in HG‐RMECs after c‐Myc silencing (Figure 6B). Simultaneously, the results of EdU and Transwell assay and angiogenesis assay in vitro documented that HG‐induced enhanced RMEC proliferation, migration and luminal formation were neutralized when c‐Myc was silenced (Figure 6C‐E). In summary, c‐Myc silencing normalized HG‐caused RMEC angiogenesis.

### 3.7 c‐Myc promoted HDAC2 expression by binding to HDAC2 promoter

**Figure 7:**
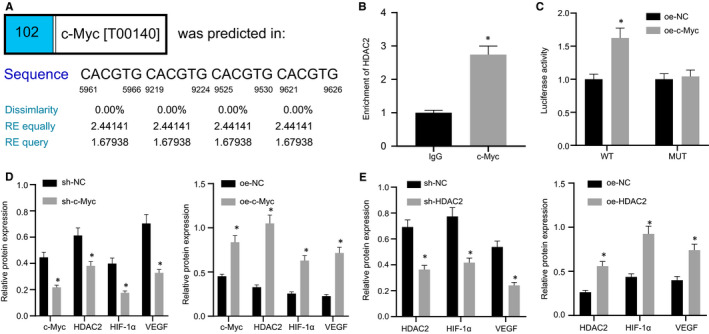
c‐Myc elevates HDAC2 expression by binding to the HDAC2 promoter. (A) Prediction of the binding site of c‐Myc to the HDAC2 promoter region using PROMO. (B) ChIP experiment to detect the binding of c‐Myc and HDAC2 promoter in RMECs. (C) Dual‐luciferase reporter assay to verify the binding of c‐Myc to HDAC2 promoter. (D) Western blot analysis of c‐Myc, HDAC2, HIF‐1α and VEGF expression in HG‐induced RMECs after overexpressing or silencing c‐Myc. (E) Western blot analysis the expression of HDAC2, HIF‐1α and VEGF in HG‐induced RMECs after overexpressing or silencing HDAC2. * *P* < .05 *vs*. treatment with IgG, sh‐NC or oe‐NC. The measurement data were expressed as mean ± standard deviation. The unpaired t test was used for comparison between two groups. The experiments were repeated 3 times

A previous study has indicated that c‐Myc binds to the HDAC2 promoter and assumes an essential role in human pluripotent stem cells, and HDAC2 was highly expressed in DR.^16^ We speculated that c‐Myc targeted HDAC2 promoter to promote angiogenesis in DR. The binding site of c‐Myc to HDAC2 promoter region were predicted using PROMO (Figure 7A). ChIP assay manifested that compared with IgG, HDAC2 enrichment was significantly augmented by overexpressing c‐Myc (Figure 7B). Dual‐luciferase reporter assay exhibited that enhanced luciferase activity in HDAC2 promoter WT subsequent to c‐Myc overexpression but unchanged luciferase activity in HDAC2 promoter MUT, suggesting that there was a direct interaction between c‐Myc and HDAC2 promoter (Figure 7C). Additionally, we found that silencing c‐Myc triggered decline in c‐Myc, HDAC2, HIF‐1α and VEGF expression in HG‐induced RMECs, and that the reverse results were noted subsequent to overexpression of c‐Myc in HG‐induced RMECs (Figure 7‐D). Thus, c‐Myc promoted HDAC2 expression by binding to the HDAC2 promoter. It is documented that HDAC2 maintains the stability of HIF‐1α ^21^ and that HIF‐1α promotes angiogenesis in DR by up‐regulating VEGF.^20^ We observed the effect of HDAC2 on HIF‐1α and VEGF by silencing or overexpressing HDAC2. HDAC2, HIF‐1α and VEGF expression in HG‐induced RMECs was obviously diminished by sh‐HDAC2 treatment, which was opposite when HDAC2 was overexpressed (Figure 7E). Collectively, c‐Myc facilitated the angiogenesis of HG‐RMECs by binding to HDAC2 promoter.

### 3.8 c‐Myc silencing blocked HDAC2/HIF‐1α/VEGF axis to repress angiogenesis in DR mice

**Figure 8:**
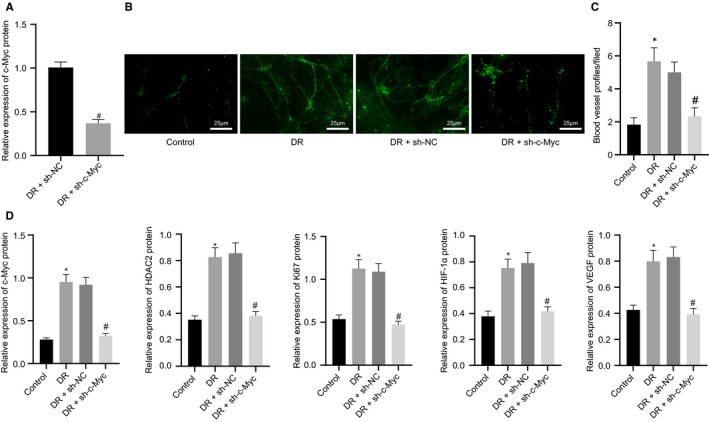
FBXW7‐mediated ubiquitination degradation of c‐Myc regulates the HDAC2/HIF‐1α/VEGF axis to inhibit angiogenesis in DR mice. (A) RT‐qPCR detection of the silencing efficiency of c‐Myc in the retina of DR mice. * *P* < .05 *vs*. sh‐NC‐treated DR mice. Mice were fed with normal diet as control, whereas DR mice were untreated, or treated with sh‐NC or sh‐c‐Myc. (B) Representative image of CD31 fluorescence staining of intraretinal angiogenesis (Arrows indicate blood vessel profiles [BVPs], scale: 25 μm.). (C) Quantitative results of panel B. (D) Western blot analysis of c‐Myc, HDAC2, Ki67, HIF‐1α and VEGF expression in the mouse retina. * *P* < .05 *vs*. normal diet‐fed mice; # *P* < .05 *vs*. DR mice treated with sh‐NC. The measurement data were expressed as mean ± standard deviation. The unpaired t test was used for comparison between two groups, and Tukey's test‐corrected one‐way ANOVA was used for comparison among multiple groups. n = 6 mice/group

We further studied the role of c‐Myc in retinal vascular dysfunction in vivo through the injection of AAV to silencing c‐Myc in the DR mouse retina. As depicted in RT‐qPCR results, notable c‐Myc down‐regulation in DR mice was triggered after sh‐c‐Myc treatment (Figure 8A). CD31 fluorescence staining results suggested that sh‐c‐Myc improved the retinal blood vessel profile, but declined RMECs entering the vitreous cavity together with normal vascular arrangement and retinal angiogenesis (Figure 8B,C). Western blot analysis displayed diminished HDAC2, HIF‐1α, Ki67 and VEGF expression in DR mice silencing c‐Myc (Figure 8D). To sum up, c‐Myc silencing inhibited DR angiogenesis in vivo by disrupting HDAC2/HIF‐1α/VEGF axis.

## 4 DISCUSSION

DR is the most common microvascular complication in diabetes, characterized by visible microvascular changes including retinal ischaemia‐reperfusion injury, inflammation, abnormal permeability, new blood vessel formation and macular oedema, which are the main causes of blindness in diabetic patients.^22^, ^23^ In recent years, evidence has shown that FBXW7 has important clinical and biological significance in the orchestration of DR,^12^ but the molecular mechanism of its orchestration of DR is not clear. Therefore, in this research, we attempted to rule out the molecular mechanism of FBXW7 in the angiogenesis of DR. Collectively, our data unveiled the inhibitory effect of FBXW7 on the angiogenesis of DR through HDAC2 by elevating ubiquitination of c‐Myc.

First, we verified the low expression of FBXW7 and the high expression of Ki67, HIF‐1α and VEGF in the retinal tissues of DR mice and HG‐induced RMECs. Consistently, the down‐regulation of FBXW7 has been detected in DR samples and hRECs in hyperglycaemia.^12^ Down‐regulation of HIF‐1α was demonstrated to suppress levels of pro‐inflammatory cytokines in DR.^24^ As reported by a previous research, the production of VEGF in DR could cause pathological angiogenesis, so treatments targeting VEGF has been widely used as novel approaches to treat such disease.^25^ Retinal angiogenesis is characterized by the proliferation, migration and tube formation of RMECs, which promotes DR.^26^ Retinal angiogenesis is closely related to the increase of HIF‐1α level, and down‐regulation of HIF‐1α decreases VEGF expression and prevents angiogenesis in a time‐dependent manner.^20^ Besides, we found that down‐regulated FBXW7 could augment angiogenesis in the retina of normal mice, while overexpression of FBXW7 could depress angiogenesis in the retina of DR mice and repress the proliferation, migration and angiogenesis of HG‐induced RMECs. Coincided with our results, evidence revealed that FBXW7 ectopic expression diminished cell angiogenesis in breast cancer by blocking the HIF‐1α‐VEGF‐A axis.^27^ Also, a research conducted by Shao et al elucidated that FBXW7 up‐regulation contributed to depression of hREC migration, proliferation and angiogenesis in DR.^12^ Further, it has shown that overexpression of FBXW7 can reduce cell proliferation, migration and lumen formation in oral squamous cell carcinoma.^28^

In the following experiments, the interaction between FBXW7 and c‐Myc has been investigated with the results revealed that FBXW7 diminished the angiogenesis of DR mice and HG‐induced RMECs and reduced HG‐induced RMEC proliferation and migration by promoting the ubiquitination degradation of c‐Myc. It was well established that FBXW7 acted as a main ubiquitin ligase for c‐Myc to promote c‐Myc ubiquitination degradation and subsequently lowered its expression in adult T cell leukaemia/lymphomas cells.^29^ It has been reported that the degradation of c‐Myc is usually ubiquitination degradation, and FBXW7 can mediate the ubiquitination of c‐Myc [9, 10]. c‐Myc is a well‐known nuclear oncoprotein that has multiple functions in cell proliferation, apoptosis and cell transformation.^30^ For example, Daga *et al* discovered the depressed migration and proliferation of cisplatin‐resistant bladder cancer cells after down‐regulating c‐Myc.^31^ Also, another research elaborated that c‐Myc overexpression contributed to elevation of tongue squamous cell carcinoma cell proliferation and migration.^32^ Similarly, overexpressed c‐Myc also could elicit the promotion of angiogenesis in renal cell carcinoma.^33^ These findings indirectly supported the contributing impact of c‐Myc on RMEC proliferation, migration and angiogenesis observed by our research. Notably, a prior study unravelled the pivotal participation of c‐Myc in DR progression by augmenting the release of Müller cell‐derived pro‐inflammatory cytokines.^15^

Another pivotal finding in our study was that c‐Myc enhanced HDAC2 expression by binding to its promoter to accelerate RMEC migration, angiogenesis and proliferation. Consistently, a study implemented by Bhandari *et al* elucidated that c‐MYC bound to HDAC2 promoter region to up‐regulate HDAC2 expression in human multipotent stem cells, thereby facilitating cell proliferation and differentiation.^16^ Moreover, enhanced HDAC activity complicated with abnormal protein acetylation has been demonstrated to be correlated with retinal degenerations related to ischaemia and ocular hypertension.^34^ From previous evidence, HDAC2 can maintain the stability of HIF‐1α expression ^22e^. Interestingly, HIF‐1α expression can promote the angiogenesis of DR by up‐regulating the expression of VEGF.^20^ Thus, we believed the interaction between HDAC2 with HIF‐1α and VEGF in DR.

**Figure 9:**
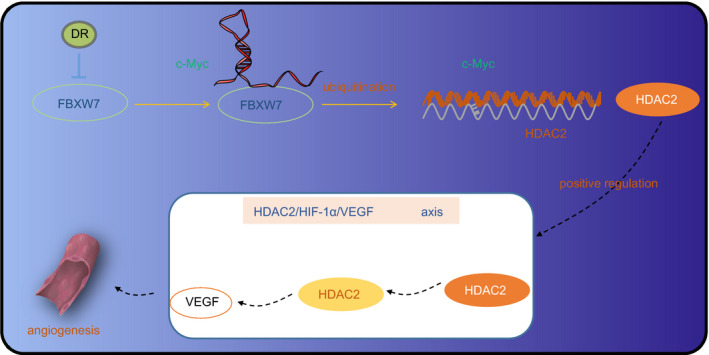
FBXW7 inhibits angiogenesis in DR via HDAC2/HIF‐1α/VEGF axis by promoting the ubiquitination degradation of c‐Myc

From the all above‐mentioned findings, we concluded that FBXW7 inactivated the *HDAC2*/*HIF‐1α*/*VEGF* axis to suppress angiogenesis in DR by elevating the ubiquitination degradation of c‐Myc (Figure 9). This study revealed that FBXW7‐mediated ubiquitination degradation of c‐Myc plays an important role in the regulation of DR, providing a sufficient theoretical basis for the study of the corresponding therapeutic target for DR. However, whether c‐Myc plays a major role in the upstream of *HDAC2* gene requires further experimental verification.

## CONFLICTS OF INTERESTS

All authors declare that they have no conflicts of interests.

## AUTHOR CONTRIBUTION

**Lihua Hu:** Conceptualization (equal); Data curation (equal); Methodology (equal); Supervision (equal); Writing‐review & editing (equal). **Xiangyun Lv:** Conceptualization (equal); Formal analysis (equal); Software (equal); Visualization (equal); Writing‐review & editing (equal). **Dai Li:** Conceptualization (equal); Data curation (equal); Investigation (equal); Resources (equal); Writing‐review & editing (equal). **Wanping Zhang:** Investigation (equal); Methodology (equal); Resources (equal); Software (equal); Writing‐review & editing (equal). **Guangyao Ran:** Formal analysis (equal); Resources (equal); Supervision (equal); Writing‐original draft (equal). **Qingchun Li:** Investigation (equal); Methodology (equal); Resources (equal); Supervision (equal); Validation (equal); Writing‐original draft (equal). **Jun Hu:** Data curation (equal); Resources (equal); Software (equal); Visualization (equal); Writing‐original draft (equal).

## Supporting information

Fig S1

Fig S2

## Data Availability

The data that support the findings of this study are available from the corresponding author upon reasonable request.
